# Moving between positions: a qualitative study of mentoring relationships in chronic eating disorders

**DOI:** 10.1186/s40337-024-01007-x

**Published:** 2024-05-16

**Authors:** Roni Elran-Barak, Shiran Elmalah-Alon

**Affiliations:** Faculty of Social Welfare and Health Sciences, University of Haifa, 199 Aba Khoushy Ave, Mount Carmel, Haifa, 3498838 Israel

**Keywords:** ED (Eating Disorders)

## Abstract

**Purpose:**

Eating disorders (ED) are chronic and challenging-to-treat conditions, often persisting over extended periods. Some patients with EDs require prolonged intensive rehabilitation services, which may include weekly home visits by paraprofessional mentors serving as support persons, providing guidelines, emotional care, and assistance. This study aims to enhance our understanding of the nature of the relationship formed between mentors and patients with EDs.

**Design & Methods:**

Fifteen in-depth qualitative interviews were conducted with patients and paraprofessional mentors. Interviews were analyzed using a descriptive phenomenological approach by two researchers to enhance reliability.

**Findings:**

Qualitative analysis highlighted that mentors play a crucial role in patients’ rehabilitation. Mentors adapt two positions (investigative and embracing) to address the encountered difficulties, including conflicts, tension, and confusion associated with (in)equality and reciprocity in the mentoring relationship.

**Practice Implications:**

Paraprofessional mentors can assist people with ED in their rehabilitation process. To support patients with EDs effectively, it is crucial to train and supervise the mentors in navigating between roles and dealing with boundaries, secrets, lies, and exposure to various aspects of the rehabilitation process. Additionally, we recommend further research using quantitative and empirical tools to complement the qualitative findings presented.

**Supplementary Information:**

The online version contains supplementary material available at 10.1186/s40337-024-01007-x.

## Impact and implications

Paraprofessional mentors can make a crucial contribution to the rehabilitation of patients with eating disorders.

Eating disorder mentors have a complex task in learning how to move between the embracing and the investigative position.

The mentor’s physical entry into the patient’s home is critically significant, as it enables an encounter typified by less concealment.

It is important to make such mentoring services available to more patients with eating disorders.

It is advised to develop a special training program for mentors, so that they can acquire knowledge and learn about the specific dynamics that are common among patients with eating disorders.

**Supplementary Information:**

The online version contains supplementary material available at 10.1186/s40337-024-01007-x.

## Abstract (plain language summary)

Eating disorders (ED) can be challenging to treat, often requiring long-term intensive support. Paraprofessional mentors are support persons who make weekly home visits to help patients in the rehabilitation process.

The study aims to understand the relationship between mentors and patients with ED. Researchers interviewed 15 patients and mentors. They discovered that mentors play a key role in helping patients get better by taking on two important roles. First, mentors act like investigators, trying to understand the challenges and problems that patients face. Second, mentors act as supporters, offering comfort and assistance during difficult times.

The study emphasizes the importance of including mentorship in ED treatment and rehabilitation. It also suggests that training and supervision for paraprofessional mentors are crucial to help them handle their roles effectively, especially when dealing with boundaries and complex situations.

**Supplementary Information:**

The online version contains supplementary material available at 10.1186/s40337-024-01007-x.

## Introduction

Eating disorders (ED) are a group of complex and life-threatening psychiatric disorders manifested in disturbed body image and maladaptive eating patterns. They include, among other things, extreme diets, fasting, binge eating, the use of laxatives, and induced vomiting [10, 25]. The most common EDs are anorexia nervosa, bulimia nervosa, and binge EDs, all of which can have a lengthy and chronic course that has a profound influence on patients’ health and well-being [15, 21]. In the most severe EDs there is – in addition to pathological eating patterns and disturbed body image – reduced psychosocial functioning, which may include difficulties in obtaining an education, preserving employment, and maintaining a social life [28]. Multidisciplinary rehabilitation that includes medical, nutritional, emotional, and familial care is essential in order to address the various aspects of the EDs [4, 19]. In-home paraprofessional mentoring care is provided to some patients with severe EDs, but studies focusing on this type of care are scarce.

In-home mentoring takes place in the natural environment of the patient and, as such, is characterized by direct non-hierarchal personal contact and collaborative work [3, 8, 13, 22–24]. Originally designed for individuals with severe mental illness who struggle to navigate life independently within the community [9], home-based mentoring offers personal support and guidance across various aspects of life, such as employment, education and social interactions. In the context of EDs, mentors hold multifaceted responsibilities. Not only do they address and support patients in establishing healthy and regular eating habits, but they also extend their focus to other essential life domains. Many patients with EDs who participate in a mentoring program also participate in regular psychotherapy sessions, and some additionally consult with a dietician. The mentoring serves as an additional treatment modality, aiding patients in managing their day-to-day tasks, thereby enhancing the overall recovery and rehabilitation process.

There are very few studies whose focus is on mentoring in EDs [3, 8, 13, 22–24]. In some, the focus has been on peer mentoring (i.e., by a peer with an ED), and in some, including the current study, the focus has been on mentoring by a non-professional (e.g., psychology students who are still not licensed to work as mental health providers). A systematic review [8] exploring the benefits, effects, and experiences of mentoring on those with EDs suggests that mentoring has great value for both mentors and mentees. The results of a qualitative study [13] focusing on mentoring among mentees undergoing intensive ED medical treatment, followed by participation in a mentoring program, revealed several benefits for mentees. These advantages included feelings of hope, reconnection with others, and increased engagement with the world. The majority of mentees emphasized the exceptional and irreplaceable support provided by their mentors. Ending the mentoring relationship at the conclusion of the program emerged as a significant challenge, with mentors citing boundary management as a primary obstacle.

Research on mentoring indicates that many fundamental challenges present in therapeutic relationships, such as building trust, fostering a sense of security, demonstrating non-judgmental attitudes, empathy, and understanding, are also relevant within mentoring relationships [3, 13]. In the context of EDs, distinctive challenges emerge, notably when attempting to address ED behaviors such as increasing caloric intake, which patients often perceive as disrupting their perceived “perfect solution” [30]. In fact, any intervention challenging the patient’s eating habits can trigger opposition, refusal to cooperate, and even false reports of eating behaviors [31]. The aim of this study was therefore to expand the body of literature addressing the unique bond formed between mentors and patients with EDs by investigating both mentors’ and patients’ personal subjective experience within this mentoring relationship.

## Methods

### Sample and procedure

The sample included 15 Israeli women, comprising ten patients and five paraprofessional mentors (as described in Tables 1 and 2). Sampling was stopped when data reached saturation [14], that is when no new information or insights are obtained from the data collected. This occurs when the researcher repeatedly encounters the same themes, concepts, or patterns in the data, and further data collection is unlikely to provide additional understanding or perspectives. The mentoring program was structured as the second phase of a rehabilitation program tailored for individuals with EDs who have finished an intensive rehabilitation program [16] and transitioned to independent living. The mentees had weekly sessions with a psychotherapist and a dietician in addition to the mentoring sessions. The mentors were supervised by the psychotherapist and/or the dietician. The aims of the mentoring program were negotiated and agreed upon between the patient, the mentor, the psychotherapist, and the dietician. The paraprofessional mentors were mostly students (Table 2). They were recruited for the mentoring position through a job positing published by the rehabilitation organization. This organization was also responsible for mentor supervision. Mentors mostly viewed this position as a part-time job allowing them to gain therapeutic skills while completing their formal mental-health education.

**Table 1:** Patients’ characteristics

Name	Age	Marital status	Children	Occupation	Education	Time with a mentor	Years with the illness	Diagnosis	Recovery Score^1^
Adi	23	Single	None	Shopkeeper	High School	4 months	6	AN	9
Nofar	34	Married	None	Mentor	College	3 years	18	BN	6
Maayan	31	Married	None	Student	Graduate School	3 years	13	AN	10
Liat	27	Single	None	Sales Clerk	College	2 years	7	AN	9
Shahar	34	Single	None	Teacher	Graduate School	2.5 years	22	AN	6
Reut	25	Married	None	Student	High School	1 year	9	AN	7
Merav	33	Single	None	Teacher assistant	College	4.5 years	23	AN	8
Jasmin	34	Single	None	Group instructor	College	2 years	11	BN	9
Maya	23	Single	None	School assistant	High School	9 months	4	AN	8
Inbal	29	Single	None	Kindergarten teacher	High School	1.5 years	18	BED	7

^1^ Participants were asked to rate their recovery level on a scale from 10 (fully recovered) to 1 (worse state ever).

**Table 2:** Mentors’ characteristics

Name	Age	Marital status	Children	Education	Time as mentor	Number of patients
Iris	29	Single	None	College	1.5 years	2
Agam	29	Single	None	College	2 years	6
Linoy	26	Single	None	College	1 year	3
Naomi	34	Married	2	Graduate School	3 years	5
Romi	27	Married	None	College	10 months	2

The research was approved by the authors’ university ethics committee (#2066). Participants were recruited to the study through an organization that provides rehabilitation services. The second author reached out to the organization’s director to request the names of potential mentors and mentees. Subsequently, the author contacted these individuals to explain the study procedure. To ensure free and independent participation, the program director was not informed about the participants’ decisions to participate or decline involvement in the study. Participants were not compensated by the research team for the interview time. All participants signed an informed consent prior to the interviews.

### The qualitative interview

The qualitative interviews were conducted in Hebrew, face-to-face (at patients’ homes or a coffee shop), in 2019 by the second author, and each lasted about 90 min. In accordance with the phenomenological method, a semi-structured interview was conducted, which focuses on questions that describe participants’ experience and their cognitive perceptions regarding the subject of the study [5]. The interview began with the following statement: *“Please describe your experience in the mentoring relationship.”* Next, participants were asked specific questions regarding different aspects, including the nature of the mentoring relationship and the role of the mentor in the rehabilitation process. The complete interview guidelines have been attached as a supplement, but the interviewer (second author) had the flexibility to navigate between questions to facilitate a natural flow in the conversation.

### The qualitative analysis

We analyzed the interviews using a descriptive phenomenological approach [7, 18]. Todres’ [26] concept of phenomenological qualitative analysis was used to reveal the essential general structure or structures of meaning within the shared experiences of the research participants. This process is conducted via bracketing: attempting to reduce the influence of researchers’ prior knowledge on the analysis [27]. To address biases, and to enhance reliability, the transcripts of the interviews were analyzed independently by the two coauthors [1]. Throughout the analysis, we systematically set aside our preconceived notions, beliefs, and presumptions regarding the subject matter. This process allowed us to engage with the data openly without imposing our prior knowledge. Following individual analyses, a collaborative discussion was conducted. We aimed to ensure that the outcomes truly reflected the shared experiences of all participants, thus enhancing the credibility and trustworthiness of our study. This joint discussion was held to integrate the results and to choose the quotations that best represented the shared experience of all participants. Another independent reader also read our analyses and approved them.

## Findings

The four themes presented below describe how the mentor’s physical entry into the patient’s home is perceived as having critical significance in the rehabilitation and recovery process. We describe the mentoring relationship as a unique opportunity for advancing the rehabilitative process (first and second themes), while highlighting the challenges associated with the mentor’s presence in the patient’s home (third and fourth themes). All four themes demonstrate the two positions that the mentors adopt: The investigative and the embracing positions. These positions emerged as key findings, identified through our analysis of the interviews, and were labeled as a result of our thorough examination of the data.

### 1. First theme: home-based care as an opportunity for a dialogue about “what’s on the table”

Interviewees emphasized that the mentor’s presence in the patient’s home allowed the exposure of eating habits and symptoms they had been hiding for a long time (“what’s on the table”). Sometimes, the straightforward act in which the mentor opens the patient’s refrigerator can be perceived as a moment of intimacy and a breakthrough during which the mentor can gain information about the patient’s eating regimen.

#### Naomi 1(mentor)

*We enter their home, in the full sense of the word, their life, their home. Unlike for example the dietitians, where it’s the patients who come to the dietitians and tell them at the clinic how it went this week […], you [the mentor] can open her [the patient’s] fridge to see if there are diet products inside.*

In the following citation, the mentee and mentor cook together. The quate demonstrates how the mentor moves between the investigative and the embracing positions and suggests that this transition can be quick. The patient, in this case, devotes herself to the act of learning and she is willing to give up previous habits, even if doing so is difficult and threatening.

#### Shahar (patient)

*I took the egg out of the fridge and when I had to put it in the mixing bowl, a little spilled out. She [the mentor] asked me if [spilling] was intentional or unintentional and to be honest I didn’t know the answer … So she told me to “pay attention” […] and then I came to get the oil and she told me I needed a tablespoon of it. For an omelet! That’s crazy… and I wouldn’t agree to it […] She really kind of taught me that the oil should cover the whole bottom of the pan … after that I wanted to pat the omelet with absorbent paper … because it was really shiny and greasy-looking. I asked her if I could, even though I knew she wouldn’t let me, but I tried my luck.*

In the following description Maayan describes a joint cooking experience with her mentor. There is a feeling, in the quote, of a mother and daughter cooking together. Maayan appreciates the mentor’s knowledge but even more than that, she appreciates the specialness and uniqueness of this joint cooking experience.

#### Maayan (patient)

*Practically, I actually also learned to cook with the help of the mentor. I still keep a recipe book I made with one of the mentors …. and it was really like the two of us were thinking together about what I felt like eating, finding a recipe and experimenting with it, so it was, like, a very meaningful experience for me.*

The following quote demonstrates how the mentor’s containing and caring presence (embracing position) allows Liat to confront her “pasta issue.” With the mentor’s help, Liat can allow herself to experience the difficulty and loss of control, but can also collect herself and confront the anxiety that arises in her when she eats pasta.

#### Liat (patient)

I had anxiety about pasta for years. It’s something I would never eat… and every time we [the mentor and I] wanted to make pasta, it ended up being put off […] So we went out one evening to eat pasta … we planned it. And it was insanely hard, I had a panic attack. I said to her, “I’m getting on a bus, I’m going home…, this is all too much for me,” and she really managed to get my panic attack under control, but I couldn’t eat the pasta. Then, we realized we might have jumped the gun: going out to eat pasta in a restaurant. When we made the pasta at home, […] and I knew what was in it, and I made it, it was a little bit easier. A month later we finally did go out to eat, in a restaurant, and I was more able to deal with it ….

The following quote demonstrates how the investigative position can help identify problematic areas that need to be addressed.

#### Romi (mentor)

*The mentor comes inside the girl’s house, assesses the situation, notices the little details. For example, I suddenly noticed with one of my patients that she had a beer mug on the counter. I asked her about it and she gave me some vague answer. After a while she told me about some beer she drank […] Because I went in, and because I know what’s going on with her, I was actually able to see and stop the drinking routine before it evolved into something bigger.*

Maya, for her part, uses the term “spotlight” to describe the role of the mentor. That is, the mentor’s intervention is experienced as a kind of strong beam of light that shines in a certain direction: the direction of recovery. The mentor illuminates the path of recovery, mentions the challenges Maya needs to overcome, and presents matters in a healthy language, a language of recovery.

#### Maya (patient)

*She [the mentor]is in my life to put a spotlight specifically on my ED. It’s very easy to forget this spotlight sometimes […] she [the mentor] is there to remind me that the thoughts that drive people [with an ED] are not the same thoughts that drive people without an ED.*

In conclusion, this theme demonstrates how the mentor’s presence in the patient’s home enables the breaking of concealment habits and promotes rehabilitative work aimed to extinguish behaviors resulting directly from the ED. Rehabilitation is made possible through the mentor’s moving back and forth between an embracing position and an “investigative” position that heeds details, casts doubt, and asks difficult questions.

### 2. Second theme: home-based care as an opportunity to work on “beside the table” issues

In contrast to the first theme, which revolved around aspects related directly to eating (“on the table”), in the second theme we see how the mentor’s presence in the house allows the mentor to witness the ED’s effect on areas of life that are not directly related to food (“beside the table”). For instance, in the next quotation, the experience of shopping with the mentor is described as an example of intimacy.

#### Inbal (patient)

*The mentor is in my life at the most personal level. She comes inside the house, she sees how I really live. Ummm, I know . I think she knows my life best. And she sees me in a variety of situations… situations in which others don’t see me. I can tell the dietitian certain things about myself, but she doesn’t experience what it’s like to actually go buy bras with me or go shopping with me and see what I have trouble with.*

In addition to shopping, another “beside the table issue” is basic hygiene. In the next quote, the mentor’s flexibility, and the transition she makes between being an authority figure and being a partner, seem to create an opening for the formation of an intimate bond.

#### Agam (mentor)

*Some houses were tough to enter, like one in which there were severe hygiene issues. It was challenging; I’d feel the need to clean up after our sessions, take a shower, and launder my clothes. There was garbage strewn around, which puzzled me. Our mentoring goals included establishing a hygiene routine; she struggled to shower regularly. We set rules, like ensuring that she showered before our meetings. We had intimate conversations; hygiene touches personal spaces deeply. We discussed menstruation and hygiene neglect. I guided her, even showing her how to wash her legs thoroughly. These were unique situations I’d never experienced.*

In the following quote we can see the mentor’s position as an investigator who reveals hidden information that no one had previously been aware of: The mentor identifies suicidal actions that would not have been identified in any other therapeutic situation. The role of the patient is to plant clues, and the role of the mentor is to reveal that which is hidden by paying attention to details.

#### Agam (mentor)

*I once found a rope on her desk, and it turned out that she slept with it tied around her neck at night. This is something that would be missed in conversation with the dietitian if she [the patient] chose not to share it. And it’s not that she shared it with me. I just saw it because I was there.*

Liat’s next quote expands on the mentor’s role as an investigator. The patient won’t reveal her secrets if no one asks questions; however, when the mentor asks the right questions, the patient will stop hiding. She’ll answer the mentor’s questions, even if they are difficult.

#### Liat (patient)

*One time when she [the mentor] came to my house, she saw that I was taking a shower in the dark. […] she just happened to notice. No one else noticed. My roommate didn’t notice; in the rehabilitation house they didn’t notice. I had always showered in the dark; this was not something new… she [the mentor] had a conversation with me about it, [I was surprised that] it didn’t seem to be something strange or unheard of to her. And we started working on it […] I had to start showering with the light on, and she [the mentor] would sit outside […] I think that when she gave me [permission to be myself]… without judgment and without criticism, I allowed myself to reveal the darkest, strangest, most illogical things associated with my ED.*

In the following two quotes, both the mentor and the patient agree that very little escapes the mentor’s eyes; she notices all sorts of behaviors. Liat uses the term “eyes in the back” to describe the situation in which the mentor is vigilant and observing all patients’ behaviors. Naomi provides a specific example - excessive shopping that the patient conceals from her psychotherapist but not from her mentor, as the mentor can see the new items when she visits the mentee.

#### Liat (patient)

*You feel that you are seen; you feel that no matter what you do, the mentor will take notice […] There should be someone there who has eyes in the back of her head. Someone who knows how to read situations, who is very alert, who knows by the tone of your voice on the phone what is happening.*

#### Naomi (mentor)

*The mentor can show her [the patient] things she does not see herself […] For example, let’s say someone had a bad day and [to make herself feel better] she went shopping and spent a thousand shekels […] Now, the patient won’t bring this subject up in any conversation because it’s not related to food; it’s not something she’s going to report. And there’s no social worker looking at her expenses. So it’s the mentor who has to put it together and talk about it with the patient.*

Nofar summarizes the mentoring experience and claims that without the mentor’s support, she could not have had a successful rehabilitation.

#### Nofar (patient)

*There’s no chance I could have succeeded in the outside world without someone watching out for me, guarding me, setting boundaries for me. I really needed them to do these things for me […] There’s no chance I would have been able to do all these things alone. No chance.*

In conclusion, the quotes illustrate the theme of “seeing,” according to which the mentor observes the patient closely, and the patient perceives the gaze as benevolent, one that helps promote recovery. In this sense it can be said that the mentor-patient relationship is a kind of dance between the two: the mentor can be both an investigator and a benevolent mother, and the patient allows the mentor to understand “what is happening.” Together they work toward rehabilitation in all life areas.

### 3. Third theme: confrontations, secrets, and lies

In the third theme, cases are depicted in which the patient feels anger toward the mentor. The mentor requires the patient to undergo a rehabilitative process and give up her ED behaviors, but the patient, in some cases, feels that she is not yet able to make these changes. In the following quote, the difficulty of bringing a mentor into the home, opening up, and devoting oneself to the rehabilitative process is described. This difficulty can later cause the patient to engage in secrets and lies.

#### Adi (patient)

*At first, I thought I might not want the experience of having a mentor, and maybe I wouldn’t need one. Like, it’s a little hard to accept the idea that you’re going to have a mentor twice a week, and I thought that maybe I could manage on my own.*

Unsurprisingly, patients often have ambivalent feelings toward the in-home mentor. Alongside moments when they want the mentor’s assistance, there are moments when they would prefer to be left alone. The mentor’s examining gaze can be perceived as threatening, and at times patients find it difficult to go along with the expectation of change and rehabilitation.

#### Romi (mentor)

*There are times when it’s difficult for them [the patients] – particularly at times when they don’t want me to see their symptoms, they don’t want my help, they prefer to be “inside” their ED, alone. There are times when they come to me and say, “I don’t have an ED, so why are you watching me?” On the other hand, there are the times they say, “Wait a minute, you’re here to help me and I need this help and I do want you to watch and eat with me and tell me if I’m eating poorly.”*

The ED can be so ingrained, and so strong, that patients will try to preserve certain behaviors, even if doing so means lying, keeping secrets, and concealing things from the mentor.

#### Agam (mentor)

*A patient will end up lying about the amount she’s really supposed to eat. Or let’s say she’s supposed to have a chocolate drink or a cup of coffee in addition to a snack, and she’ll say, “No, it’s only in the morning” or “It’s only at ten” or “I’ve already drunk or already eaten,” and all sorts of such things. Or there are girls who will say, “I’ll do it later” or “I’ll do it tomorrow,” and you just know they won’t […] You’ll either close your eyes to it and [ignore it] and say, “Okay, I believe she’ll do it tomorrow” or you’ll [confront her] but say it in a tone of: “I want to help you, to be with you, let’s do it together, let’s make it easy on you.”*

When the mentor enters the patient’s home, the patient’s ability to hide is at least partially taken away from her, making her feel she has no choice but to find other ways to hide the things she is afraid to reveal. The following quote shows the patient’s insight into her behaviors:

#### Liat (patient)

*There are many things that are hidden from sight, that are very difficult to see if you do not bring them up … Let’s put it this way: I wouldn’t want to be a mentor to someone with an ED … it’s … it’s hard, it’s hard. You have to be very sharp to see that … not everything is fine […] I think you have to keep your eyes wide open because we are manipulative and liars… […] We know how to make people believe every word that comes out of our mouths and turn everything around in a way [.] that’s safe for us. But this is not necessarily good for us. I think you [the mentor] must absolutely be skeptical of everything we tell you.*

The next quote describes how despite the confrontations, anger, and lies, the mentoring relationship can eventually be used to promote rehabilitation.

#### Shahar (patient)

*There’s no way even to cheat a little bit, or to fool the mentor. Yesterday at dinner I made myself a toasted cheese sandwich and she [the mentor] said, before I had even put the sandwich in the sandwich maker, “Remember, do not squash it down too much. Just put the top down and close it; so the cheese doesn’t come out of the toast” [.] So when she said to me, “Remember not to do it,” I immediately said, “I didn’t even want to do that! Why are you putting ideas in my head??” Like I got mad at her… but that wasn’t right of me to get mad at her, because actually I probably did plan to do what she said, and that’s why I got annoyed that she said it […] mentoring is intense, and it’s just the two of you and the mentor gets to know your “tricks” very quickly.*

In conclusion, the above quotes highlight the inevitable conflicts that arise in the mentoring relationship. It seems clear that this relationship has to withstand complex challenges of lies and concealment, and that the ability of the mentor and mentee to overcome these challenges is critical in the recovery process.

### 4. Fourth theme: home-based care as an acknowledgment of inequality - similar but different

Sometimes patients try to see the mentor as a friend or older sister, thus blurring the hierarchy that exists between them. In the following quote we can see Jasmine’s difficulty in accepting the built-in inequality of the mentor-patient relationship.

#### Jasmine (patient)

*A lot of my difficulties with Shirley [the mentor] stemmed from the fact that it was an unequal relationship. I wanted to know more about her ]…[ It was hard for me because I felt like, because of the situation we were in, she couldn’t see that I too had abilities. And also there were moments when I didn’t want to express any particular weaknesses because I wanted to be with her in some equal place. Because there’s something really weird about it. It’s true that she’s not my age, she’s older than me by a few years, but it doesn’t matter, I have girlfriends who are her age, I have older girlfriends. We often had a lot of laughs, and it was funny and fun for us, like on a human level, we already knew each other so well that it was like … She knew about my difficulties, she knew about my weaknesses, about my strengths. I kept telling her about me, but I seemed to know nothing about her…*.

In contrast to Jasmine who experienced difficulties with the inequality, Reut described the advantage of the mentor’s “in-between” position.

#### Reut (patient)

*I think it’s good to have someone who’s somewhere between being a professional and being a …. I don’t know how to define it… a more mature person who can accompany you during the process […] a friend who was older than me and in a different place in life. Um … there is something about this in-between situation […] the lack of formal professional training allows for something a little more fluid and natural. It feels like the help [from the mentor] is also more at the everyday level […] things are more practical .*

The tension around the unequal mentor-patient status can also put the mentor in uncomfortable and challenging situations. Linoy ranged from wanting to set a personal example for the patients to wanting to be authentic (e.g., eating the amount of food that actually suited her). Linoy made sacrifices in order to create in the patient a feeling (even a deceptive one) of equality in the relationship.

#### Linoy (mentor)

*I don’t really have the option of coming and telling the patients I’m not hungry. [When we sit down for meals] I should be able to eat the same amounts they’re supposed to eat; I can’t eat less than what they’re supposed to eat. There is some minimum I need to eat, so I eat it, there’s nothing to be done about it. I try to plan my day accordingly and not show any difficulty [when I’m with them]. If I have a hard time eating, they’ll definitely notice; girls with EDs are very sensitive to these little nuances. So, of course, they watch me too. It bothers me. First of all it bothers me because I’m eating alongside them and it feels a bit intrusive sometimes: They look at what I eat, and whether I eat properly, and whether I’m different [from the patient]; it is a bit intrusive. On the other hand, it’s also my job. I’m supposed to come and set an example for them and if I don’t set an example for them, it’s a bit problematic in my opinion.*

In many cases, patients try to overstep boundaries and invade the mentor’s privacy; they also test the relationship in ways that could threaten it. As can be seen below, this issue intensifies when Agam wonders whether, in different circumstances, she and the patient could have been friends.

#### Agam (mentor)

*The girls I worked with were around my age, studying social work or education. We frequented similar places, had mutual acquaintances, and even worked in similar settings. One of the girls started asking me personal questions from the very first session—not extremely personal, but enough for me to set clear boundaries. She’d comment on my Facebook photos, discussing whether they were flattering or not, asking about my weight, my past, and my diet. It wasn’t easy. I had to firmly tell her not to ask such questions, to clarify that we weren’t going to discuss my weight, that I wasn’t her girlfriend. It took time for us to adjust to each other. Eventually, with most of the girls, I felt it was successful. However, some of them felt like they could have been my little sister in another universe. There were moments when I could have had fun with them, but it was challenging initially. I had to understand what triggered me in their presence and quickly discern what was my own and what was theirs.*

Along these same lines, sometimes there was tension between mentor and patient regarding perceptions of the relationship’s boundaries. Merav’s below quote highlights the confusion that was created when the mentor angrily dismissed Merav’s interest in her personal life.

#### Merav (patient)

*My mentor had moved apartments and she talked about her move. [After a few days] I asked her how the move went because I care about her and I was interested, because I really love her. […] But she was very angry with me. She said she didn’t want to talk about it, and that she was the one who would decide whether to talk about it […]and it wasn’t pleasant for her … and I told her that it felt like she had something against me, that she had a hard time with me. So if she was having a hard time with me, then let’s talk about it.*

Iris, a mentor, talked about how carefully the boundaries between mentor and patient must be negotiated.

#### Iris (mentor)

*But I realized over the years that in order to make contact, in order to get them to trust me, I would need to bend the boundaries a bit at first. Like, I would need to be a bit warm with them or speak to them from a place of equal standing, so that later they would listen to me and give me respect.*

In conclusion, this theme demonstrates how the closeness between mentor and patient can create tension and sometimes even role confusion: There must be an acknowledgement of the similarities between them but also of the essential difference. Many mentors aim to meet this challenge by setting clear boundaries while also allowing, at times, moments of flexibility and boundary crossing (e.g., when the mentor shares something personal about her life with the mentee) in order to maintain a sense of partnership and intimacy.

## Discussion

**Fig. 1:**
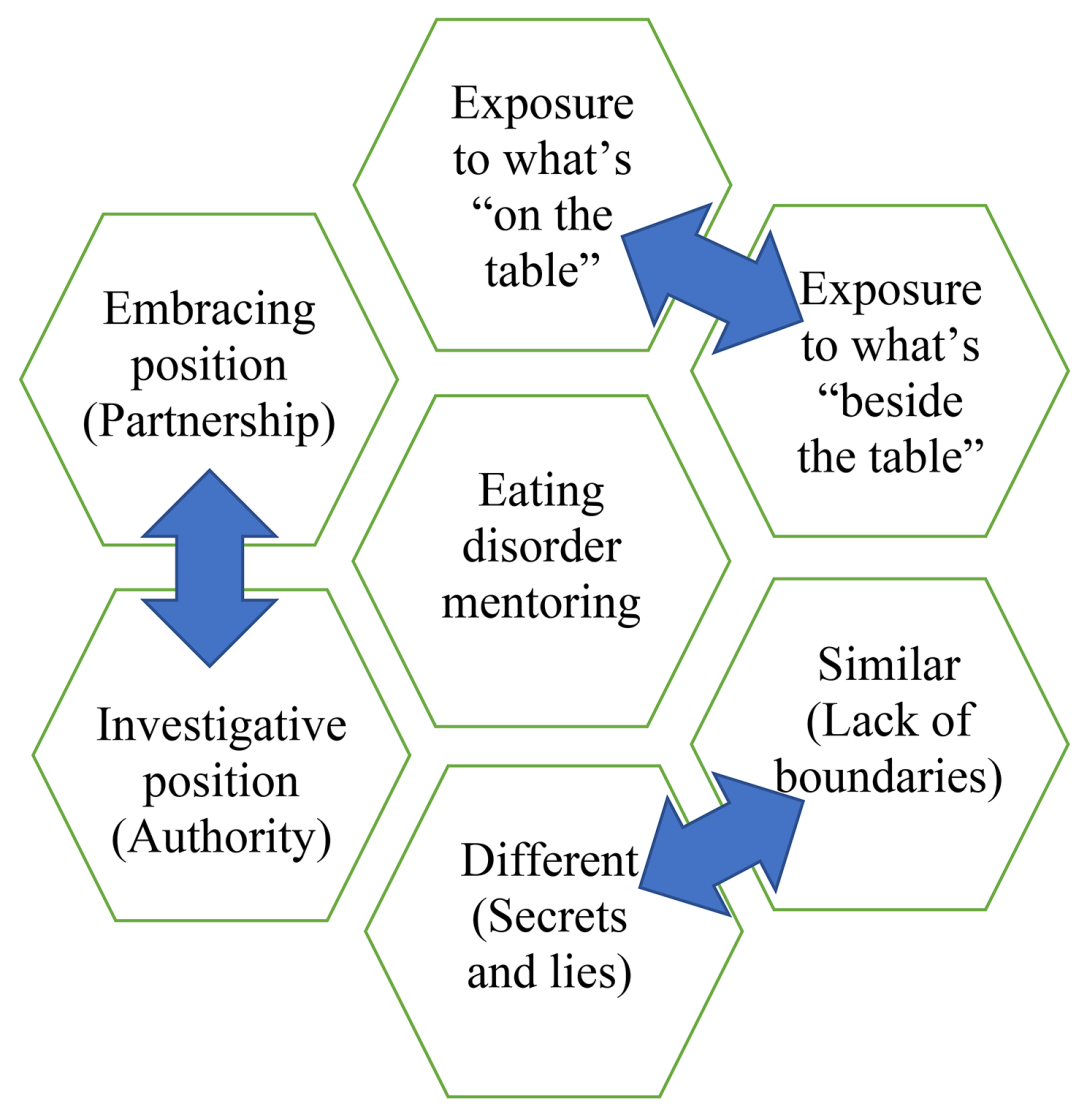
Home-based care for eating disorders - When the mentor enters the patient’s home

This qualitative study was designed to examine the unique experience of in-home mentoring relationships in the context of EDs, as perceived by both mentors and patients. Four themes emerged, all of which describe the unique bonding experience between the two, and its contribution to the rehabilitation process. Mentors and patients alike emphasized that the mentor’s physical entry into the patient’s home is critically significant, as it enables an encounter typified by less concealment, during which the mentor can gain access to the patient’s hidden and intimate world. Figure 1 demonstrates the complex task of the ED mentor who enters the patient’s home and must learn to move between the embracing and investigative position while dealing with boundaries, secrets and lies, and exposure to what’s both “on” and “beside” the table.

Patients with EDs tend to be secretive about their symptoms [2, 12]. They tend to avoid sharing the difficulties they face while at the same time feeling they are “alone in the world” and cannot trust other people, including therapists, especially due to the fear and shame of being exposed to judgment. In addition, as some of the ED symptoms (e.g., fasting, vomiting, over-exercising) serve the patients’ goal (i.e., losing weight), they would rather conceal these symptoms than have the therapist try to eradicate them [2, 20]. Therefore, secrecy and lack of self-disclosure are perceived in the literature as one of the major challenges faced by ED therapists [2, 12]. In this context, the mentoring relationship, which allows the mentor to come inside the house and see what’s “on the table” can be helpful in overcoming concealment issues that oftentimes prevent ED treatments from succeeding.

Prior findings from qualitative interviews about mentoring relationships in the context of EDs have highlighted the concealment barrier [17] – that is, when patients avoid revealing information about themselves to their mentors. It is possible that patients fear that mentors will criticize and even reject them [20] and may worry that once mentors know what is really happening, they will put pressure on patients to change their eating habits (e.g., add more food to their meals) [11]. In a retrospective study [29], it was suggested that secretive behaviors were planned and conscious strategies among most (57-73%) patients with EDs. These secretive behaviors include attempts to conceal the truth, such as providing incorrect information about what has been consumed, or refusing to get on the scale during therapeutic sessions. It has also been suggested that therapists’ attempts to discover these secretive behaviors are followed by patients’ negative reactions [29]. Our findings suggest that the mentor’s entrance into the patient’s home can on the one hand be experienced as intrusive and threatening (as explained in the third theme), but at the same time can break the vicious cycle of secrecy (as explained in the first and second themes) and promote recovery. The mentoring relationship takes place in the midst of a conflict in which patients both wish to receive help but also fear the consequences of their exposure. If mentors are able to behave in a consistent and empathic way, while being sensitive to patients’ internal conflict (i.e., moving between the embracing and the investigative position, according to the patient’s needs) both parties will presumably be able to overcome this barrier and help patients reveal what they are going through.

Another major challenge in the mentoring relationship is the issue of boundaries. Clear boundaries, for instance regarding the role of each member, are crucial in helping mental health professionals define, determine, and maintain the therapeutic relationship [6]. The mentor-patient relationship in this context suffers from an inherent lack of clarity about the nature of the distance between the parties – for example, when patients invade mentors’ privacy by looking at the mentors as friends and expecting them to reveal details about their personal lives (as explained in the fourth theme). Our findings suggest that each mentor must find her own individual way of defining boundaries in this context, and therefore mentors would benefit from close supervision to help them tailor and establish boundaries for the benefit of their patients.

Overall, the study findings highlight important aspects of mentors´ tasks and roles in the context of severe and enduring EDs. Some of these tasks and roles overlap with those of other mental health providers (e.g., dieticians, psychotherapists), while others are specific to the mentors. Both mentors and other ED professionals must learn how to move between the “embracing” position, in which they hold a containing and caring presence, and the “investigative” position, in which they identify problematic areas that need to be addressed. However, the mentoring relationship is unique because certain boundaries may easily dissolve, requiring mentors to put extra effort into their establishment. The mentoring relationship takes place in the mentee’s home, and the mentor can therefore be perceived as a visiting friend, in contrast to the experience that takes place in a formal therapeutic setting typically found in a clinic. This “informal” setting demands that mentors navigate the delicate balance between intimacy and professionalism and work diligently to establish clear boundaries that might otherwise easily dissolve. It is also possible that the mentor and mentee will be of similar ages or backgrounds, adding depth to the mentoring bond but also creating a challenge to the professional relationship. Given that mentors work in a team, and often receive supervision from the psychotherapist or the dietician who regularly sees the patient, it is vital for all team members to align on treatment goals and collaborate effectively toward achieving them.

Despite the contribution of this qualitative study to the field, several limitations should be noted. Although saturation was achieved, it is possible that participants who were not included in this study (e.g., patients who refuse receiving assistance from mentors) would have shed light on factors other than the ones presented. We would recommend that researchers, in further studies, examine the mentoring relationship via the use of quantitative and empirical tools to supplement the findings presented. Such studies could include standardized surveys assessing mentor-mentee dynamics, structured observation protocols during mentoring sessions, and outcome measures quantifying specific aspects of recovery or progress.

The findings of this study have important implications for practice. First, it is critical to draw attention to the significant contribution that mentoring relationships can make to patients with EDs, and it is important to make this service available to more patients. That said, mentors are not licensed mental health professionals; they therefore need to have a great deal of knowledge about mental health rehabilitation in general and EDs in particular. They must also have several personal strengths including sensitivity, empathy, persistence, and assertiveness. These personal strengths will help them overcome the main challenges that are experienced, according to our findings (Fig. 1), in home-based mentoring relationships. It is important to develop a special training program for mentors, so that they can acquire knowledge and learn about the specific dynamics that are common among patients with EDs. In addition, clinical supervision is required so that mentors can bring up their mentor-patient dilemmas with mental health professionals (e.g., clinical social workers) along the way. Due to the nature of EDs, mentors’ dilemmas (e.g., boundaries, secrecy) can be complex, and mentoring work in this context can put a great deal of pressure on mentors that can be both stressful and cumulative. Mentors need to be able to reflect on their dilemmas in the presence of a clinical supervisor, as it may be almost impossible for mentors to find solutions on their own, and without continuous clinical supervision.

### Electronic supplementary material

Below is the link to the electronic supplementary material.

Supplementary Material 1

## Data Availability

Data and study materials are available upon request from the corresponding author.

## Footnotes

1 Pseudonyms are used for all participants.
