# Supplementary material for: Moving between positions: a qualitative study of mentoring relationships in chronic eating disorders
Source: J Eat Disord. 2024 May 16;12:59. doi: 10.1186/s40337-024-01007-x (PMC11097531; doi:10.1186/s40337-024-01007-x)
Supplement: Supplementary file 1 — Supplementary Material 1 [file 40337_2024_1007_MOESM1_ESM.docx]

1. The role of the mentor:
   - How do you perceive the mentor's role in EDs?
   - What significance does this role hold for you?
   - How does this position contribute within a rehabilitation program, compared to other roles of healthcare professionals?
   - What skills do you consider essential for this role?
   - What aspects would you preserve and improve regarding the mentor's role?
   - Share aspects of your experiences that you are proud of.
   - Reflect on your meeting with the mentor.
   - Considering mentoring doesn't require formal professional training, how does this impact the relationship? In what ways?
2. Relationships promoting recovery:
   - How does your relationship with the mentor aid your recovery? Can you provide an example?
   - Share aspects of your relationship with the mentor that you are proud of.
   - Share aspects of your relationship with the mentor that you are not proud of.
   - Assess your confidence level in the relationship with the mentor.
   - Discuss the significance of the relationship with the mentor in your recovery journey.
3. Aspects of eating disorders:
   - From your perspective, what qualities of the mentor support the recovery process in EDs?
   - What challenges did you face when establishing contact with the mentor, and how did you address them?
   - What are the fundamental aspects you value in your ED journey with the mentor?
   - Describe your feelings regarding your relationship with your mentor in relation to your ED symptoms.
4. Conclusion of the interview:
   - Are there any additional topics you believe are crucial to understanding the mentor relationship's contribution to the recovery process?
